# WHAT CAN COUNTY-LEVEL EMERGENCY MEDICAL SERVICES DATA TEACH PUBLIC HEALTH ABOUT OLDER ADULT FALLS

**DOI:** 10.1093/geroni/igz038.1852

**Published:** 2019-11-08

**Authors:** Carolyn R Ham, Xinyao deGrauw, Scott Dorsey

**Affiliations:** 1 Washington State Department of Health, Washington, United States; 2 Snohomish County Fire District 7, Snohomish County, Washington, United States

## Abstract

Fall-related injuries in older adults contribute to an increasing number of deaths, hospitalizations and Emergency Medical Services (EMS) responses. In Snohomish County, the third largest county in Washington State, EMS agencies with electronic health records cover 96% of the county’s population. This EMS data contains unique information on falls, which can estimate costs and clarify intervention priorities. We analyzed 2018 data from EMS in Snohomish County. Fall incidents were summarized by count, frequency, and rate per 1,000 population. Costs for transferring patients to emergency departments (ED) were estimated using 2015 Snohomish Community Paramedic Analysis, and direct medical costs averted by implementing a single intervention were estimated based on prior research by Stevens and Lee (2018). There were total 38,910 incidents in older adults, of those 4,777 incidents were caused by falls (1606 in males and 2906 in females). The mean age (SD) was 81.0 (±8.9). The incidence rate was 45.6 per 1000 (55.8 in females and 30.6 in males). There were 573 repeated falls (12%). Most of the falls happened at home (54.85%), followed by assisted living and nursing homes (27.84%). 85.53% of the falls were transferred to ED, at an estimated cost of 3.15 million dollars. We calculated that one million dollars in medical cost could be averted by implementing home modifications delivered by an occupational therapist (OT). This research demonstrates the utility of EMS data for describing fall injury and determining interventions. Fall prevention programs should focus on preventing repeated falls and addressing home safety risks.

